# Immunomodulation of inflammatory leukocyte markers during intravenous immunoglobulin treatment associated with clinical efficacy in chronic inflammatory demyelinating polyradiculoneuropathy

**DOI:** 10.1002/brb3.516

**Published:** 2016-07-14

**Authors:** Wayne B. Dyer, Joanne C. G. Tan, Timothy Day, Lynette Kiers, Matthew C. Kiernan, Con Yiannikas, Stephen Reddel, Karl Ng, Phillip Mondy, Peta M. Dennington, Melinda M. Dean, Halina M. Trist, Cristobal dos Remedios, P. Mark Hogarth, Steve Vucic, David O. Irving

**Affiliations:** ^1^ Australian Red Cross Blood ServiceAlexandriaNSWAustralia; ^2^ Sydney Medical SchoolUniversity of SydneyCamperdownNSWAustralia; ^3^ Cabrini Medical CentreCabrini HospitalMalvernVic.Australia; ^4^ Department of NeurophysiologyRoyal Melbourne HospitalParkvilleVic.Australia; ^5^ Brain and Mind CentreUniversity of SydneyCamperdownNSWAustralia; ^6^ Burwest NeurophysiologyBurwoodNSWAustralia; ^7^ Department of NeurologyConcord Repatriation and General HospitalConcordNSWAustralia; ^8^ Department of NeurophysiologyRoyal North Shore HospitalSt LeonardsNSWAustralia; ^9^ Australian Red Cross Blood ServiceKelvin GroveQldAustralia; ^10^ Burnet InstitutePrahranVic.Australia; ^11^ Department of NeurologyWestmead HospitalWestmeadNSWAustralia; ^12^ University of TechnologySydneyNSWAustralia

**Keywords:** autoimmune neuropathies, chronic inflammatory demyelinating polyradiculoneuropathy, dendritic cells, disease pathways, Fc‐gamma receptors, immunophenotyping, intravenous immunoglobulin G

## Abstract

**Objective:**

The objective of the study was to profile leukocyte markers modulated during intravenous immunoglobulin (IVIg) treatment, and to identify markers and immune pathways associated with clinical efficacy of IVIg for chronic inflammatory demyelinating polyradiculoneuropathy (CIDP) with potential for monitoring treatment efficacy.

**Methods:**

Response to IVIg treatment in newly diagnosed IVIg‐naïve and established IVIg‐experienced patients was assessed by changes in expression of inflammatory leukocyte markers by flow cytometry. The adjusted INCAT disability and Medical Research Council sum scores defined clinical response.

**Results:**

Intravenous immunoglobulin modulated immunopathogenic pathways associated with inflammatory disease in CIDP. Leukocyte markers of clinical efficacy included reduced CD185^+^ follicular helper T cells, increased regulatory markers (CD23 and CD72) on B cells, and reduction in the circulating inflammatory CD16^+^ myeloid dendritic cell (mDC) population and concomitant increase in CD62L and CD195 defining a less inflammatory lymphoid homing mDC phenotype. A decline in inflammatory CD16^+^ dendritic cells was associated with clinical improvement or stability, and correlated with magnitude of improvement in neurological assessment scores, but did not predict relapse. IVIg also induced a nonspecific improvement in regulatory and reduced inflammatory markers not associated with clinical response.

**Conclusions:**

Clinically effective IVIg modulated inflammatory and regulatory pathways associated with ongoing control or resolution of CIDP disease. Some of these markers have potential for monitoring outcome.

## 1 Introduction

Chronic inflammatory demyelinating polyradiculoneuropathy (CIDP) is an immunologically heterogeneous autoimmune inflammatory disease resulting in peripheral nerve demyelination, causing profound disability in more than 50% of cases (Franssen & Straver, 2014; Mathey et al., 2015). High‐dose intravenous immunoglobulin (IVIg), with a superior safety profile to steroids (Dalakas, 2012), is a preferred first‐line treatment with established evidence of efficacy (Hughes et al., 2008). The kinetics of response to IVIg in CIDP is similar to plasma exchange (Dalakas, 2012) and is faster than that reported for corticosteroids (Van den Bergh et al., 2010). Short‐term prednisolone or pulsed dexamethasone may induce long‐term remission (Eftimov, Vermeulen, van Doorn, Brusse, & van Schaik, 2012), whereas IVIg does not eliminate the underlying cause of disease and ongoing IVIg is required to prevent relapse. Individual optimization of IVIg dosing is required because of individual rates of IgG catabolism in order to achieve a pharmacokinetic profile supporting treatment efficacy (Kuitwaard et al., 2013; Rajabally, Wong, & Kearney, 2013). To accommodate patient‐specific metabolism of IVIg, early high‐dose IVIg is recommended for initial recovery followed by titration to the minimal effective dose (Mathey & Pollard, 2013), whereas low‐dose initial treatment may be insufficient to prevent irreversible axonal loss in some CIDP variants (Dalakas, 2011, 2012; Mathey & Pollard, 2013). However, there are no definitive strategies to predict the optimal IVIg dose nor the response to treatment, suggesting frequent monitoring is required to establish optimal treatment in new patients.

Immunomodulatory effects of IVIg have been observed across diverse leukocyte populations, including immunopathogenic disease pathways characteristic of CIDP (Dalakas, 2011; Ephrem et al., 2005; Mathey & Pollard, 2013; Mathey et al., 2015). Based on preliminary phenotyping of activated leukocytes cultured with IVIg, we hypothesized that biomarkers of efficacy of IVIg may be found on multiple leukocyte populations detected in peripheral blood. This study reports immunophenotyping of major leukocyte populations aimed at identifying changes in leukocyte surface marker expression during IVIg treatment. Markers altered by IVIg treatment were then tested for association with clinical efficacy. The data provide evidence that immune phenotyping before and after IVIg treatment can be used to distinguish responders from nonresponders, and suggest targeting‐specific markers and pathways for monitoring clinical response to IVIg in CIDP is feasible.

## 2 Methods

### 2.1 Ethical review

Recruitment of CIDP patients in New South Wales was approved by the Sydney Local Health District (RPAH Zone) HREC, and in Victoria by individual institutional HRECs. All study participants gave informed consent.

### 2.2 CIDP patients

Newly diagnosed (IVIg‐naïve) patients (*n* = 7) and established (IVIg‐experienced) patients (*n* = 14) were examined over the course of two consecutive IVIg treatment cycles. Diagnosis was by clinical and electrophysiological criteria (typical or atypical, sensory and motor, pure motor, or sensory ataxic) according to European Federation of Neurological Societies/Peripheral Nerve Society guidelines (Van den Bergh et al., 2010). Inclusion and exclusion criteria for newly diagnosed CIDP were as used for the ICE trial (Hughes et al., 2008), excepting patient N9 with sensory CIDP. One patient (E14) received low‐dose prednisolone (12 mg/day) and methotrexate (20 mg/week); no other patient received steroids.

### 2.3 IVIg treatment

The two treatment cycles were included, according to patient group: (1) induction and initial maintenance cycles in newly diagnosed patients and (2) two maintenance cycles in patients on established dose‐titrated regimens. Standard‐of‐care IVIg induction dosing was 2 g/kg over 3–5 consecutive days; individually optimized maintenance doses ranged from 0.4 to 1 g/kg every 2–4 weeks according to the Australian guidelines (National IVIg Criteria Review Working Group, 2012).

### 2.4 Blood collection and clinical assessment

Blood samples were collected prior to IVIg infusion (day 0) and on day 7 during each cycle, and leukocyte marker expression was quantified by flow cytometry. Clinical scores were measured at the start and end of each treatment cycle. For the adjusted INCAT disability score (Hughes et al., 2001), a clinically meaningful change was defined as one point (change between 0 and 1 for upper limb function was not clinically significant in the adjusted INCAT scale). For the Medical Research Council (MRC) sum score (Kleyweg, van der Meche, & Schmitz, 1991), a two‐point change was determined to be clinically meaningful. Response in newly diagnosed patients was defined as at least 1 INCAT point improvement during the induction cycle and stable or improved in the second. Patient N9, with a predominantly sensory deficit, was also assessed by the INCAT sensory sum score (Merkies, Schmitz, van der Meche, & van Doorn, 2000). Patients responding to established IVIg regimens were scrutinized for response versus relapse during individual treatment cycles, according to stable versus a clinically meaningful deterioration in either neurological score as defined above.

### 2.5 Full blood counts and flow cytometry panels

Full blood counts were performed on a CellDyn Ruby automated counter (Abbott Diagnostics, Lane Cove, NSW, Australia) using EDTA‐anticoagulated blood. Whole blood flow cytometry was performed on heparinized blood. Antibody clones chosen for the leukocyte markers are listed in Table 3, and all flow cytometry reagents were sourced from BD Biosciences (North Ryde, NSW, Australia) unless indicated. A large number of markers were screened in order to identify potential changes in population and functional markers, with the expectation that only a few markers would have changes detected in peripheral blood. Flow cytometry was performed by two scientists at a single site using the same flow cytometer and standardized procedures. Antibodies were incubated with blood (100 μL/test) for 15 min before treating with FacsLyse. Tubes for intracellular markers in Treg cells were treated with FacsPerm, washed, and incubated with antibodies for 30 min. Washed leukocytes were run on a BD FACS Canto II, with appropriate compensation settings. Raw.fcs files were analyzed with Flow‐Jo software (Tree Star Inc, Ashland, OR, USA), gates were set against appropriate isotype controls. Marker expression refers to % positive cells in the parent leukocyte population.

### 2.6 Statistical analysis

Statistical analysis was performed using Prism software (GraphPad, San Diego, CA, USA). An initial screen of markers influenced by IVIg treatment was determined by the Wilcoxon signed‐rank test, with significance defined by *p* < .05. Markers that changed after IVIg treatment were associated with clinical efficacy after segregating data from responder versus nonresponse/relapse cycles, reporting mean ± *SD* and paired *t* tests to determine the magnitude of change in marker expression associated with clinical efficacy. Correction for multiple markers associated with clinical efficacy was applied within each leukocyte population. Difference in maker expression before IVIg treatment between response and relapse cycles was determined by two‐tailed Mann–Whitney test. Association between change in marker expression and clinical outcome used Fisher's exact test. Association between the magnitude of change in marker expression and change in neurology scores was tested by Spearman's rank correlation coefficient.

## 3 Results

### 3.1 Clinical response to IVIg treatment

On the expectation that clinical response could be determined in new CIDP patients after only two IVIg treatment cycles (Hughes et al., 2008), the two initial treatment cycles were used to determine clinical response defined by the disability scores. Two consecutive treatment cycles from patients on established IVIg regimens were studied to compare marker responses with new patients, to determine the stability of markers of clinical response, and to identify markers associated with potential episodes of clinical relapse. Patient details, IVIg regimens, and clinical scores for each treatment cycle are listed in Table 1. Clinically effective IVIg treatment was recorded in 11 of 17 treatment cycles in newly diagnosed and 27 of 32 cycles in established patients. Isolated cycles characterized by clinical relapse were recorded, suggesting that some IVIg doses may have been at the threshold of clinical efficacy in some patients. Clinical response to each treatment cycle was not associated with IVIg dose, pretreatment variables including disability scores, or leukocyte counts (Table 2), although mean lymphocyte count tended to be higher in established patients that relapsed.

**Table 1:** Patient details, intravenous immunoglobulin (IVIg) dose, and neurological response for each treatment cycle

Patient	Age	Gender	IVIg regimen^a^		Neurological response to IVIg^b^				Observed relapse or nonresponse
			Cycle 1	Cycle 2	Clinical score	Pre‐IVIg	Post cycle 1	Post cycle 2	
Newly diagnosed (IVIg‐naïve) patients									
N1	43	F	2.0/4 W	ND	NCAT^c^	3	2	ND^d^	
					MRC	52	57		
N2	70	M	2.0/4 W	0.4/4 W	INCAT	9	5	5	
					MRC	44	44	44	
N3	56	M	2.0/4 W	0.4/4 W	INCAT	5	3	**4** d	Relapse cycle 2
					MRC	60	60	60	
N4	56	F	2.0/4 W	0.4/4 W	INCAT	3	2	1	
					MRC	55	58	56.5	
N5	78	M	2.0/3 W	1.0/3 W	INCAT	5	**5**	**5**	Nonresponse
					MRC	54	**54**	**54**	
N6	78	M	2.0/3 W	1.0/3 W	INCAT	7	2	2	
					MRC	42	56	56	
N7	65	M	2.0/3 W	1.0/3 W	INCAT	5	**5**	4	Cycle 1 nonresponse
					MRC	54	**54**	52	
N8	35	M	1.0/4 W	0.4/4 W	INCAT	2	**3**	**3**	Nonresponse
					MRC	59	**59**	**56**	
N9	75	M	2.0/4 W	0.4/4 W	INCAT	1	1	1	
					MRC	60	60	60	
					INCAT‐S	8	7	6	
Established (IVIg‐experienced) CIDP patients									
E1	76	F	0.2/4 W	0.4/4 W	INCAT	2	2	1	
					MRC	56	56	58	
E2	68	F	1.0/3 W	1.0/3 W	INCAT	3	3	3	
					MRC	52	52	54	
E3	52	M	0.7/4 W	0.7/4 W	INCAT	2	1	**2**	Relapse cycle 2
					MRC	60	60	60	
E4	77	F	0.4/4 W	0.4/4 W	INCAT	1	1	0	
					MRC	58	60	60	
E5	67	F	0.9/4 W	0.9/4 W	INCAT	6	6	6	Relapse cycle 2
					MRC	44	44	**41**	
E6	67	M	0.4/4 W	0.4/4 W	INCAT	2	2	**4**	Relapse cycle 2
					MRC	60	60	60	
E7	70	M	0.8/4 W	0.8/4 W	INCAT	4	3	3	
					MRC	46	60	60	
E8	76	M	0.4/4 W	0.4/4 W	INCAT	6	5	5	
					MRC	42	52	56	
E9	67	M	0.8/3 W	0.8/3 W	INCAT	2	2	2	
					MRC	56	57	58	
E10	73	F	1.0/3 W	1.0/3 W	INCAT	2	2	2	
					MRC	44	46	46	
E11	37	F	1.0/3 W	1.0/3 W	INCAT	3	3	3	
					MRC	52	54	58	
E12	80	M	0.4/4 W	0.4/4 W	INCAT	2	2	2	
					MRC	54	56	56	
E13	66	M	0.4/2 W	0.4/2 W	INCAT	3	3	3	Relapse cycle 1
					MRC	54	**50**	52	
E14	72	M	0.7/3 W	0.7/3 W	INCAT	3	3	3	Relapse cycle 2
					MRC	54	54	**52**	
E15	70	M	0.4/4 W	0.4/4 W	INCAT	3	3	3	
					MRC	59	59	59	
E16	52	M	0.4/4 W	0.4/4 W	INCAT	2	2	1	
					MRC	60	60	60	

ND, second treatment cycle not given.

a IVIg dose (g/kg) and cycle length in weeks (W).

b Neurological scores that defined relapse or nonresponse are given in bold.

c INCAT disability score (0–10; 0 = normal function), MRC sum score (max. 60 = normal function), and INCAT‐S (sensory sum score) (0–20; 0 = normal function).

d Missing data: N1 withdrew from the study after the first cycle and blood samples were not collected from the second cycle of N3.

**Table 2:** Pretreatment variables, disability scores, and leukocyte counts were not associated with clinical response

Pretreatment variables	Newly diagnosed (IVIg‐naïve) CIDP			Established (IVIg‐experienced) CIDP		
	Response^a^	Nonresponse^a^	*p* a	Response	Relapse	*p*
IVIg cycles (*n*)	11	6		27	5	
IVIg dose	1.35 ± 0.73	1.35 ± 0.79	1.000	0.59 ± 0.26	0.61 ± 0.22	.880
Steroid use		Nil		(Patient E14: prednisolone 12 mg/day)		
Age at treatment	66.1 ± 9.4	69.3 ± 10.8	.641	68.0 ± 11.6	64.8 ± 7.5	.415
INCAT disability	4.6 ± 2.4	4.5 ± 1.0	.903	2.8 ± 1.3	3.0 ± 1.9	.815
MRC sum score	51.6 ± 7.1	55.5 ± 3.0	.216	53.0 ± 5.5	54.4 ± 6.6	.692
Lymphocytes (10^9^/L)	1.77 ± 0.32	1.64 ± 0.64	.713	2.03 ± 0.81	3.07 ± 0.95	.070
Monocytes (10^9^/L)	0.54 ± 0.13	0.71 ± 0.48	.522	0.64 ± 0.45	0.79 ± 0.53	.580

a Mean ± *SD* and Mann–Whitney test.

### 3.2 Leukocyte markers influenced by IVIg treatment

The effect of IVIg treatment on circulating leukocyte populations was screened across a wide range of surface antigens representing both subpopulation and functional markers. Multiple redundant markers were tested because it was not known which would be detectable in peripheral blood during an in vivo response to IVIg, compared to our preliminary data from cultured leukocytes. For example, changed expression of CD25, CD38, CD69, CD71, and CD95 were identified after in vitro T‐cell activation and subsequent exposure to IVIg. Therefore, only one or two robust markers representing T‐cell responses, identified by the initial screen of response to IVIg, were needed for evaluation of clinical efficacy of IVIg treatment. Despite broad changes in markers representing diverse leukocyte populations in our preliminary in vitro cultures, only a few markers changed in peripheral blood sampled after IVIg treatment (Table 3). However, changes in marker expression 7 days after IVIg treatment, measured during two treatment cycles per patient, were found in each major leukocyte population. These changes included decreased follicular helper T cells (CD185^+^ CD4 T cells), increased Treg cell circulation (reduced CD184) and increased activation (HLA‐DR), and naïve B cells with increased inhibitory and regulatory markers (CD23^+^ and CD72^+^), but reduced inhibitory CD32b^+^ B cells. IVIg treatment resulted in a decrease in the inflammatory CD16^+^ myeloid dendritic cell (mDC) population and a corresponding increase in markers defining a noninflammatory mDC population (CD62L and CD195), whereas monocytes responded with reduced CD32a and CD32b expression.

**Table 3:** Leukocyte populations and effect of intravenous immunoglobulin (IVIg) treatment on surface marker expression

Leukocyte population (phenotype)	Investigational markers	Mab clone	Changed in marker expression^a^		
T cells (CD45^+^CD3^+^CD4^±^)			CD4^+^	CD4^−^	
	CD25	M‐A251	0.819	0.330	
	CD26	M‐A261	1.000	1.000	
	CD27	M‐T271	1.000	0.750	
	CD28	CD28.2	0.160	0.164	
	CD38	HIT2	0.895	0.090	
	CD69	L78	0.055	0.055	
	CD71	M‐A712	0.313	0.742	
	CD95	CX2	0.755	0.319	
	CD103	Ber‐ACT8	0.844	0.469	
	CD120b	hTNFR‐M1	0.859	0.190	
	CD184	12G5	0.335	0.516	
	**CD185** b	51505	**0.042**	0.213	Decrease
	CD195	2D7/CCR5	0.960	0.402	
	**CD197**	150503	0.282	**0.013**	Decrease
Regulatory T cells (CD45^+^CD4^+^CD25^HI^ CD127^LO^)	Total population surface:				
	CD62L	DREG‐56	0.192		
	**CD184**	12G5	**0.034**		Decrease
	CD195	2D7/CCR5	0.140		
	**HLA‐DR**	G46‐6	**0.011**		Increase
	Intracellular				
	CD152	BNI3	0.470		
	FoxP3	259D/C7	0.110		
	TGFβ	9016	0.695		
Naïve and memory B cells (CD19^+^ CD27^±^)			Naïve	Memory	
	**CD23**	M‐L233	**0.0097**	0.976	Increase
	**CD32/32b** d	FLI8.26/X63	**0.012**	**0.0004**	Decrease
	CD32a	IV	0.899	0.577	
	CD40	5C3	0.967	0.095	
	CD69	L78	0.599	0.969	
	CD70	Ki‐24	0.898	0.247	
	**CD72**	J4‐117	**0.007**	0.375	Increase
	CD80	L307.4	0.641	0.358	
	CD86	2331 FUN‐1	0.979	0.360	
	HLA‐DR	G46‐6	0.054	0.437	
Myeloid dendritic cells (CD45^+^HLA‐DR^+^CD11c^+^ lineage‐neg)^e^	**CD16**	3G8	**0.0017**		Decrease
	CD32/32a^d^	FLI8.26/IV.3	0.468		
	CD32b	X63	0.946		
	CD40	5C3			
	**CD62L**	DREG‐56	**0.0006**		Increase
	CD64	10.1	0.368		
	CD83	HB15e	0.091		
	CD86	2331 FUN‐1	0.814		
	CD184	12G5	0.995		
	**CD195**	2D7/CCR5	**0.0077**		Increase
	CD300f	UP‐D2	0.356		
Monocytes (CD45^+^CD14^+^)	CD16	B73.1	0.528		
	**CD32/32a** d	FLI8.26/IV.3	**0.0057**		Decrease
	**CD32b**	X63	**0.050**		Decrease
	CD38	HIT2	0.149		
	CD40	5C3	0.179		
	CD64	10.1	0.229		
	CD69	L78	0.632		
	CD80	L307.4	0.549		
	CD86	2331 FUN‐1	0.843		
	CD163	GHI/61	0.872		
	CD184	12G5	0.159		
	CD195	2D7/CCR5	0.547		
	CD204^b^	SR‐AI/MSR1	0.139		
	CD206	19.2	0.484		
	CD300f^c^	UP‐D2	0.077		
	HLA‐DR	G46‐6	0.547		

^a^Change in expression was determined by Wilcoxons signed‐rank test; significant changes (*p* ≤ 0.05) are in bold font.

All antibodies were from BD Pharmingen unless indicated: ^b^R&D Systems; ^c^Biolegend; and ^d^CD32 was replaced during the study with isoform‐specific CD32a and CD32b antibodies produced by HMT and PMH (Ramsland et al., 2011). CD32 expression on dendritic cells and monocytes is equivalent to CD32a, and CD32 on B cells is equivalent to CD32b, and therefore data were pooled where appropriate.

^e^Gated on cells negative for lineage markers CD3, CD14, CD19, CD20, CD34, CD56, and CD66.

The rationale to analyze both newly treated and patients on established IVIg regimens together was the need to first identify markers influenced by IVIg, and also IVIg treatment is required to maintain clinical effect through frequent modulation of pathogenic processes. After segregating responder from nonresponse/relapse data, markers associated with clinical efficacy were determined according to increased significance of association (lower *p* values) compared with the combined data or opposing direction of change in marker expression between responder and nonresponse/relapse cycles. From the 13 markers found in 7 leukocyte populations that were influenced by IVIg treatment, only 6 markers were specifically associated with clinical efficacy of IVIg treatment in CIDP (Table 4). Correction for multiple testing in these markers using Bonferroni correction within each leukocyte population resulted in an adjusted level of significance, indicated in Table 4. If a conservative correction was based on all 13 markers, the adjusted level required for significance was *p* = .0038, and only four markers remained significant (Table 4). We decided to apply Bonferroni correction within leukocyte populations, because it is immunologically implausible to classify follicular helper T cells and CD16^+^ mDCs as linked variables, whereas CD16, CD62L, and CD195 were linked markers on mDCs and required appropriate correction. These markers associated with clinical efficacy signified a change away from an inflammatory profile. But none of these markers were capable of predicting clinical outcome before IVIg treatment. Baseline CD184 expression on Treg cells was higher in nonresponders, but this marker was not specifically associated with clinical efficacy (Table 4).

**Table 4:** Effect of intravenous immunoglobulin (IVIg) treatment on leukocyte populations and markers associated with clinical efficacy in chronic inflammatory demyelinating polyradiculoneuropathy

Population	Marker	Wilcoxon test^a^	Response to IVIg (all patients)			Responders (clinical efficacy)			Nonresponse/relapse			Pre‐IVIg predictor (response vs. relapse)^c^Mann–Whitney
			Pre‐IVIg	Post IVIg	Paired *t* test^b^	Pre‐IVIg	Post IVIg	Paired*t* test	Pre‐IVIg	Post IVIg	Paired*t* test	
CD4T cells	CD185	0.042	19.2 ± 7.4	17.5 ± 7.3	0.013	19.4 ± 8.0	17.2 ± 7.9	**0.008** d	18.4 ± 4.8	18.8 ± 4.1	0.645	0.616
CD8T cells	CD197	0.013	27.7 ± 14.7	25.4 ± 14.8	0.020	27.6 ± 15.2	25.9 ± 15.9	0.120	28.3 ± 13.5	23.5 ± 10.1	0.059	0.885
Tregs	CD184	0.034	64.6 ± 19.6	59.0 ± 20.8	0.033	61.1 ± 20.8	55.9 ± 20.4	0.097	74.7 ± 12.0	68.1 ± 20.3	0.190	0.029
	HLA‐DR	0.011	33.8 ± 14.7	36.1 ± 13.5	0.016	34.0 ± 15.1	35.9 ± 13.6	1.000	33.2 ± 14.5	36.4 ± 13.9	0.026	0.914
NaïveB cells	CD23	0.010	54.8 ± 23.9	60.3 ± 19.6	0.008	54.2 ± 25.0	61.6 ± 20.8	0.0009*	56.6 ± 20.6	55.7 ± 14.8	0.884	0.811
	CD72	0.007	84.2 ± 12.9	86.1 ± 11.4	0.159	83.9 ± 13.2	87.3 ± 11.3	**0.010**	85.4 ± 12.4	86.1 ± 11.6	0.426	0.739
	CD32b	0.020	43.0 ± 20.3	36.6 ± 22.5	0.012	43.2 ± 20.4	36.3 ± 22.7	0.031	42.4 ± 19.6	37.3 ± 23.1	0.202	0.918
MemoryB cells	CD32b	0.0002	54.0 ± 21.7	44.8 ± 24.5	0.0004	55.1 ± 22.2	46.9 ± 25.4	0.002	49.9 ± 20.3	37.6 ± 20.6	0.082	0.521
Myeloid dendritic cells	CD16	0.002	57.1 ± 18.1	48.5 ± 20.9	0.0004	58.0 ± 18.4	46.4 ± 21.7	<0.00001*	53.8 ± 17.2	56.5 ± 16.0	0.433	0.500
	CD62L	0.0006	26.0 ± 14.6	32.5 ± 15.8	0.0003	27.4 ± 15.4	35.0 ± 16.7	0.0005*	20.7 ± 10.1	23.4 ± 6.7	0.315	0.117
	CD195	0.008	13.1 ± 11.1	17.5 ± 10.3	0.002	13.4 ± 10.7	18.8 ± 14.8	0.001*	12.1 ± 13.0	12.7 ± 10.3	0.823	0.777
Monocytes	CD32a	0.006	74.0 ± 30.9	67.0 ± 35.9	0.014	75.4 ± 29.8	67.8 ± 35.8	0.026	69.8 ± 35.1	64.5 ± 37.8	0.338	0.657
	CD32b	0.050	50.8 ± 38.9	40.7 ± 36.7	0.079	53.7 ± 36.9	38.1 ± 36.5	0.032	42.2 ± 47.9	48.7 ± 40.5	0.148	0.642

a Significance of paired data based on the direction of change from initial screen of IVIg response.

b Significance of paired data based on the mean difference in expression before and after treatment.

c Expression of markers before IVIg treatment were compared (responders vs. nonresponders/relapse) to identify any markers capable of predicting clinical response.

d Correction for multiple testing according to Bonferroni was based on the number of markers analyzed in each leukocyte population (two markers per population: *p* = .025; three markers per population: *p* = .017; conservative correction for all 13 markers: *p* = .0038). Bold markers were associated with clinical efficacy and passed correction at the population level; an asterisk identified markers that passed conservative Bonferroni correction.

### 3.3 Decreased CD16^+^ mDC population is associated with clinical outcome

**Figure 1:**
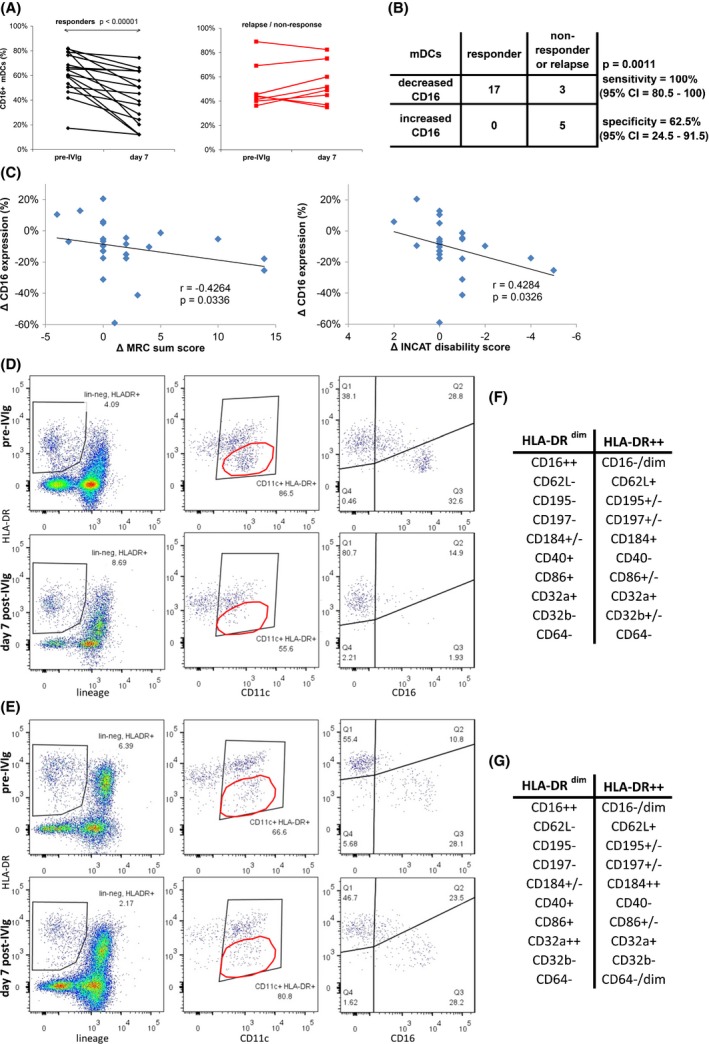
Decreased CD16^+^ myeloid dendritic cell population after intravenous immunoglobulin (IVIg) was associated with clinical efficacy. (A) Change in CD16^+^ mDCs during the treatment cycle that defined response from relapse or nonresponse in each patient. (B) Predictability of decreased CD16^+^ mDCs on clinical outcome was supported by sensitivity but not specificity (Fisher's exact test). (C) The magnitude of CD16 downregulation on mDCs correlated with increased MRC sum scores, and decreased INCAT disability scores (Spearman's rank correlation). Decreased CD16 expression was associated with loss of the HLA‐DR ^low^ CD16^+^ mDC population after IVIg in a responder patient (D), but retention of this population after IVIg in a relapsing patient (E). The HLA ^−^ DR ^low^ CD11c^+^ population is outlined by the elliptical gate in the middle panels, and the CD16/HLA‐DR gate was set according to this HLA‐DR ^low^ population and isotype control for CD16. The pre‐IVIg phenotype of this HLA‐DR ^low^ CD11c^+^ population was the same for both responding patient (F) and relapsing patient (G), which defined an activated mature but circulating inflammatory mDC population that declined after clinically effective therapy

We next investigated the suitability of these markers to provide early indication of outcome from a treatment cycle, aimed at monitoring efficacy when comparing treatment options, including dose titration. Six markers associated with clinical efficacy were observed during two treatment cycles per patient. A single treatment cycle that defined clinical outcome for each patient was selected (the relapse cycle was chosen in response‐discordant patients), and increase versus decrease in marker expression was tested for predicting clinical outcome measured at the end of that cycle (Fisher's exact test). Of these six markers, only CD16^+^ mDCs were directly associated with clinical outcome using Fisher's exact test for sensitivity and specificity (Fig. 1). Decreased CD16^+^ mDCs in responders provided sensitivity for predicting outcome (100%; 95% CI = 80.5–100), but specificity was weak (Fig. 1B), also driving down expression in some relapsing patients. However, the magnitude of decreased CD16^+^ mDCs correlated with improved neurological disability scores (MRC and INCAT; Fig. 1C). The MRC sum score defined relapse in established patients, whereas the INCAT generally defined nonresponse in new patients (Table 1).

The immunological relevance of reduced CD16^+^ mDCs was further investigated. The CD16^+^ mDC population was also characterized as HLA‐DR^low^. Stable clinical response was associated with loss of this population after IVIg, along with a reduced proportion of HLA‐DR^++^ cells expressing CD16 (Fig. 1D). Clinical relapse was associated with either no change or an increase in the CD16^+^HLA‐DR^low^ population (Fig. 1E). Phenotypic profiling of the CD16^+^HLA‐DR^low^ and the CD16^−^HLA‐DR^++^ populations suggested that clinically effective IVIg treatment decreased an activated (CD40^+^CD86^+^), nonlymphoid (CD62L^−^, CD195^−^, CD197^−^) circulating population that expressed activating Fcγ receptors CD16 and CD32a, but not the inhibitory CD32b. This CD16^+^HLA‐DR^low^ population lacked the activating FcγR CD64 associated with monocyte‐derived DCs. Although decreased after clinically effective IVIg treatment, this CD16^+^ mDC population cycled back to baseline levels measured immediately prior to the following treatment cycle. This suggests de novo regeneration of new inflammatory mDCs between treatment cycles is a possible cause of ongoing inflammation in CIDP requiring life‐long IVIg treatment.

## 4 Discussion

This study confirmed some of the known immunomodulatory action of IVIg on disease pathways of inflammatory neuropathies, and identified markers associated with clinical outcome. Changes in disease pathways associated with clinical efficacy included reduced capacity for autoantibody production associated with decreased follicular helper T cells and increased expression of regulatory and tolerance markers on B cells. A decrease in circulating inflammatory CD16^+^ mDCs was associated with clinical efficacy. In addition, markers associated with a reduced inflammatory profile, independent of clinical response, may signify the overall nonspecific anti‐inflammatory effects of IVIg which in combination may contribute to clinical efficacy. Although IVIg may induce long‐term immunomodulatory changes, including inhibitory Fcγ receptor expression, or autoreactive T‐cell populations (Klehmet et al., 2015), most of the anti‐inflammatory effects of IVIg observed in our study were short‐lived, suggesting the underlying causes of CIDP are modulated but not removed by IVIg in most patients. It is thought that the transitory effect of IVIg on clinical outcome may be a consequence of catabolism of infused IgG, impacting on direct competition with pathogenic autoantibodies and complement inactivation (Berger, McCallus, & Lin, 2013). The transitory effect of IVIg treatment on markers identified in this study may likewise reflect the decline in IVIg interactions with Fcγ receptors and the downstream regulation of B cell, mDC and follicular helper T‐cell‐mediated responses.

The phenotypic profile of the CD16^+^HLA‐DR^low^ mDC population suggests these were mature (CD195^−^) and inflammatory (CD40^+^CD86^+^), but unlike conventional mature mDCs, locked in peripheral circulation with impaired lymphoid migration (CD62L^−^CD184^low^CD197^−^; Kim & Diamond, 2015; Lutz & Schuler, 2002; Martin‐Fontecha, Lanzavecchia, & Sallusto, 2009; Sallusto et al., 1998). Furthermore, these CD16^+^HLA‐DR^low^ mDCs had receptors for inflammatory (CD16, CD32a) but not regulatory (CD32b) antibody interaction. Reduction of this CD16^+^HLA‐DR^low^ mDC population resulted in a relative increase of lymph‐node homing markers (CD62L and CD195) in the remaining CD16^−^HLA‐DR^++^‐enriched mDC population, conferring reduced inflammatory potential (Wildenberg et al., 2008). The origin of this CD16^+^HLA‐DR^low^ population is controversial, some suggesting a monocytic origin (Kim & Diamond, 2015; Randolph, Sanchez‐Schmitz, Liebman, & Schakel, 2002). However, low CD205 expression and lack of CD64 on this population suggest otherwise. Regardless of the source, accumulation between treatment cycles of a circulating activated inflammatory mDC population deficient for lymph‐node homing markers may be a major immunopathogenic pathway of CIDP disease, and depletion of this population may be a critical immunoregulatory action of IVIg. The relative increase in CD195 and CD62L defining the CD16^−^HLA‐DR^++^ mDC population after clinically effective IVIg suggests increased lymph‐node homing and reduced peripheral tissue inflammation is another immunoregulatory action of IVIg. However, lack of key homing markers on the CD16^+^HLA‐DR^low^ mDC population does not explain removal from circulation after IVIg.

B‐cell activation and generation of autoantibodies are considered critical immunopathogenic pathways in CIDP and other autoimmune neuropathies. Data from this study showed that IVIg increased both regulatory (CD23) and peripheral tolerance (CD72) markers on naïve B cells. The inhibitory Fcγ receptor CD32b was expressed on more memory B cells before IVIg treatment in responders than nonresponders, but was not directly associated with clinical outcome. CD185 expression defines follicular helper T cells with increased homing for B‐cell germinal centers and is elevated in a number of autoimmune diseases (Park et al., 2014). The observed decrease in follicular helper T cells after IVIg may reduce autoantibody production as well as macrophage activation pathways (Schmitt, Bentebibel, & Ueno, 2014; Slight et al., 2013).

Inhibitory Fcγ receptor‐mediated pathways of immunomodulation in B cells and monocytes are considered primary mechanisms of IVIg immunomodulation (Tackenberg et al., 2009), and was confirmed by data from this study. Expression of CD32b decreased after IVIg on both monocytes and memory B cells, as reported on monocytes in IVIg‐treated immune thrombocytopenia (Shimomura et al., 2012). Downregulation of CD32b on B cells may result from increased CD40 ligation (Zhang et al., 2013). Alternatively, an increase in the balance of type‐2 and regulatory cytokines induced by IVIg treatment may promote CD32b expression (Boruchov et al., 2005), and transitory CD32b downregulation observed in this study may be a temporary effect of cytokine neutralization after IVIg infusion.

In searching for biomarkers to monitor and predict outcome of IVIg therapy, an ideal biomarker should be sensitive to changes in IVIg dosing and predict clinical outcome soon after treatment, as measured 7 days after infusion in this study. Useful biomarkers should be able to guide optimal IVIg dosing for new CIDP patients, and provide early warning of relapse when performing dose tapering or testing for IVIg dependence when in remission. The six markers associated with clinical efficacy identified in this study may be suitable for monitoring therapeutic response in combination. They signified a reduced inflammatory profile and correlated with clinical outcome, albeit a temporary change for most markers, reverting toward baseline before the next IVIg infusion. A decreased CD16^+^ mDC population was associated with response to IVIg and demonstrates the feasibility of using this marker in a test to establish optimal IVIg dosing for new CIDP patients, although specificity was insufficient to predict relapse during dose tapering. Additionally, the small number of patients in this study means that these data cannot be used to guide clinical decisions on IVIg dosing. Confirmation of the critical components of these immunomodulatory pathways may facilitate development of novel therapeutics aimed at replacing this expensive and nonspecific anti‐inflammatory treatment, and such markers may provide the means to test efficacy.

## Funding Information

The study was funded by the Australian Red Cross Blood Service. The Australian Government funds the Australian Red Cross Blood Service to provide blood, blood products, and services to the Australian community.

## Conflict of Interest

None declared.
